# Resveratrol attenuates intermittent hypoxia-induced insulin resistance in rats: Involvement of Sirtuin 1 and the phosphatidylinositol-4,5-bisphosphate 3-kinase/AKT pathway

**DOI:** 10.3892/mmr.2014.2762

**Published:** 2014-10-23

**Authors:** QIUYAN WANG, XIAOHUI SUN, XIAOBIN LI, XIANG DONG, PENG LI, LI ZHAO

**Affiliations:** 1 Department of Respiratory Medicine, Shengjing Hospital of China Medical University, Shenyang, Liaoning 110001, P.R. China; 2 Emergency Department, First Affiliated Hospital of Dalian Medical University, Dalian 116011, P.R. China

**Keywords:** resveratrol, intermittent hypoxia, insulin resistance, sirtuin 1, obstructive sleep apnea

## Abstract

Obstructive sleep apnea can induce chronic intermittent hypoxia (CIH) during sleep and is associated with obesity and diabetes. Resveratrol (RSV), a polyphenolic phytoalexin, can regulate glucose metabolism, thereby reducing insulin resistance. The present study aimed to assess whether RSV attenuates CIH-induced insulin resistance in rats and the underlying mechanisms. A total of 40 rats were randomly assigned into five groups: i) Control; ii) subjected to CIH only; iii) subjected to CIH and treated with 3 mg/kg/day of RSV; iv) subjected to CIH and treated with 30 mg/kg/day of RSV; v) subjected to CIH and treated with 60 mg/kg/day of RSV. All animals were sacrificed following 28 days of treatment. Subsequently, the blood and livers were harvested and blood insulin and glucose levels were measured. Levels of sirtuin (Sirt) 1, insulin receptor (InsR) and glucose transporter 2 (Glut2) in the liver were measured. RSV treatment was demonstrated to suppress weight gain and improve hepatic morphology. RSV treatment also significantly reduced the homeostasis model assessment estimate of insulin resistance of the rats exposed to CIH. This effect occurred in a dose-dependent manner. RSV significantly upregulated liver Sirt1 levels and inhibited InsR and Glut2 expression in the liver. Additionally, RSV activated the phosphorylation of phosphatidylinositol-4,5-bisphosphate 3-kinase (PI3K) and AKT. The present study demonstrates that RSV prevents CIH-induced insulin resistance in rats. Upregulation of Sirt1 and activation of PI3K/AKT signaling may be involved in this process.

## Introduction

Obstructive sleep apnea (OSA) is a complex pathophysiological condition affecting 1–4% of the general population and 25–35% of obese individuals (1,2). It is characterized by repetitive upper airway obstruction, frequent snoring, apnea during sleep, chronic sleep loss at night and excessive daytime sleeping (3). OSA can induce chronic intermittent hypoxia (CIH) during sleep. There are established associations with obesity, insulin resistance (IR), glucose intolerance and other metabolic syndromes (4). Clinical and epidemiological studies have suggested that sleep disturbances are associated with a group of metabolic disorders, including IR, diabetes mellitus, hypertension and dyslipidemia (5–9). In particular, sleep loss is an independent risk factor affecting the development and progression of diabetes. Sleep loss contributes to IR and diabetes either by directly interfering with the regulation of glucose metabolism, or indirectly through weight gain and obesity by affecting appetite (10).

Resveratrol (trans-3,5,4-trihydroxystilbene, RSV), a natural polyphenolic phytoalexin, is highly concentrated in grapes and red wine. RSV is known to promote weight loss, reduce IR and improve disease symptoms in patients with diabetes (11,12). RSV has been hypothesized to be a potent activator of the mammalian silent information regulator gene Sirtuin 1 (*SIRT1*) (12,13). Previously, *SIRT3* and *SIRT5* were identified as direct targets of RSV (14).

Sirtuins are a group of nicotinamide adenine dinucleotide-dependent protein deacetylases (15). There are seven members in this family, including Sirt 1–7. Sirtuins are involved in numerous biological functions involving cell growth, apoptosis, energy metabolism and the stress response (16–18). Sirt1 is widely expressed in mammalian tissues. Activation of Sirt1 promotes insulin secretion, reduces glucose tolerance and decreases body weight (19,20). Animal studies have demonstrated that *SIRT1* deficiency in liver leads to hyperglycemia, oxidative damage and insulin resistance (21). Sirt1 is important in regulating energy metabolism and glucose homeostasis. Blood glucose levels are tightly regulated within a narrow range and hypoglycemia and hyperglycemia have a detrimental effect. Glucose homeostasis is critically dependent on the levels of insulin released by pancreatic β-cells and on the ability of insulin to inhibit the production of hepatic glucose, while promoting the uptake of glucose in peripheral tissues. The responsiveness of the β-cells as well as insulin sensitivity are affected by sleep (22,23). Sleep loss and IR are closely associated with OSA. RSV has been reported to reduce IR and maintain blood glucose levels within the normal range. However, there are few studies on the efficacy of RSV in treating patients with OSA. In order to investigate the potential therapeutic application of RSV for treating patients with OSA, a chronic intermittent hypoxic model in rats was generated to mimic the chronic hypoxia and IR presented in patients with OSA. The aim of the present study was to determine the efficacy of RSV in reducing CIH-induced IR in rats and the underlying molecular mechanisms.

## Materials and methods

### Animals

A total of 40 eight-week-old male Sprague-Dawley rats purchased from Chang Sheng Biotechnology Corp. (Liaoning, China) were used in the present study. Animals were housed in the animal facility at the Shenghai Hospital of China Medical University (Shenyang, China) with standard 12 h light/dark cycles and *ad libitum* access to food and water. All animal procedures were approved by the Institutional Animal Care and Use Committee of the Shengjing Hospital of China Medical University (Shenyang, China). RSV was purchased from Guanyu Biotechnology Corp. (Shanxi, China). Animals were randomly assigned into five different groups with eight rats in each group: i) Control group: animals were exposed to normal conditions without RSV treatment; ii) CIH group: animals were exposed to intermittent hypoxic conditions without RSV treatment; iii) CIH plus low RSV group: animals were exposed to intermittent hypoxic conditions and administered daily with 3 mg/kg/day of RSV; iv) CIH plus median RSV group: animals were exposed to intermittent hypoxic conditions and administered daily with 30 mg/kg/day of RSV; v) CIH plus high RSV group: animals were exposed to intermittent hypoxic conditions and administered daily with 60 mg/kg/day of RSV. RSV was prepared by adding 3 ml water. Rats were fed via oral gavage with 3 ml RSV daily, for a total of 28 days. Rats in control groups without RSV treatment were administered 3 ml of water as vehicle via an oral gavage. The body weight of animals was recorded every week for all animals. Blood glucose levels and insulin levels were recorded at the end of the experiment for all animals.

### CIH exposure

Animals were exposed to intermittent hypoxia every day for 8 h, from 9 a.m. to 5 p.m., for 28 days, as described previously (24,25). Briefly, housing cages were placed inside plexiglas chambers where the oxygen level was monitored and regulated by timer-controlled valves connected to room air source and a nitrogen source. The air and nitrogen entered the chamber via separate flow meters. During this period of time, oxygen was reduced from 21% to 10% over 1.5 min, maintained at 10% for 1.5 min, returned to 21% over 1 min and held at 21% for 2 min. This cycle was repeated for 8 h. Control animals were housed in identical chambers for an equivalent amount of time and were exposed to the same timer and valve-controlled changes in air flow as the CIH rats; however, the only source of gas in the control chambers was room air, so they remained at normoxic levels throughout the investigation. Immediately following the final hypoxia session, the cages were returned to the main housing room. The animals were fasted for 12 h prior to day 29 following the treatment and then sacrificed under isoflurane anesthesia. Liver tissues and blood samples were collected.

### Histology

Animals were sacrificed in a preprandial state under isoflurane anesthesia on day 29. Liver tissues were collected from all animals. All tissues were rapidly frozen in liquid nitrogen and stored at −80°C for future investigation. Paraffin-embedded tissue sections (4 μm thick) were obtained and were stained with hematoxylin and eosin (H&E).

### Detection of blood glucose and insulin levels

Blood glucose concentration was measured using a Contour glucometer (Bayer, Pittsburgh, PA, USA) and Contour blood glucose test strips (Bayer). Plasma insulin was determined using a Rat Insulin ELISA kit (Millipore, Billerica, MA, USA) according to the manufacturer’s instructions. The value of the homeostatic model assessment (HOMA)-IR index was calculated using the following formula: [Fasting insulin concentration (μU/ml) × fasting glucose concentration (mmol/l)] / 22.5.

### Immunohistochemistry (IHC)

Sirt1 protein expression in paraffin-embedded hepatic tissue sections was detected using a standard avidin-biotin immunoperoxidase protocol. Briefly, liver sections were deparaffinized and pretreated with 1% H_2_O_2_ for 30 min to quench endogenous peroxidase activity, followed by three rinses in phosphate-buffered saline (PBS). The samples were blocked with 10% goat serum (Solarbio, Beijing, China) and subsequently incubated with 1:50 dilution of polyclonal rabbit anti-rat Sirt1 antibody (Santa Cruz Biotechnology, Inc., Santa Cruz, CA, USA) overnight at 4°C. This was followed by incubation with 1:500 dilution of polyclonal goat anti-rabbit secondary antibody (Beyotime, Shanghai, China) at room temperature for 1 h. Immune complexes were detected using an avidin-3,3′-diaminobenzidine staining kit (Beijing Zhongshan Biotechnology, Beijing, China). The sections were counterstained with hematoxylin and mounted. Images of the slides were captured using a stereo BX51 light microscope (Olympus, Tokyo, Japan) equipped with a digital camera.

### RNA analysis and reverse transcription quantitative polymerase chain reaction (RT-qPCR)

*SIRT1* expression was determined by RT-qPCR as described previously (26). Briefly, total RNA was isolated from rat liver using TRIzol reagent (Invitrogen Life Technologies, Carlsbad, CA, USA) according to the manufacturer’s instructions. cDNA was synthesized by RT using the QuantiTec Reverse Transcription kit (Tiangen Biotech, Beijing, China). Subsequently, qPCR was performed using the following primers: *Sirt1*, forward 5′-ACCCTCAATTTCTGTTCTGC-3′ and reverse 5′-TTGGACATTACCACGTCTGC-3′; β-actin, forward 5′-GGAGATTACTGCCCTGGCTCCTAGC-3′ and reverse 5′-GGCCGGACTCATCGTACTCCTGCTT-3′. The cycle threshold (Ct) values of the target gene were first normalized to β-actin from the same sample and subsequently the relative differences between the groups were calculated and expressed as relative mRNA levels, setting the control group as 1. Each sample was assessed in triplicate.

### Western blotting

Liver tissue was homogenized in radio immunoprecipitation lysis buffer (Beyotime) supplemented with protease inhibitors (PMSF and aprotonin) and protein phosphatase inhibitor (NaF and sodium orthovandate). The protein concentration was determined using a bicinchoninic acid assay protein assay kit (Beyotime). Protein aliquots (40 μg) were separated on a 10% sodium dodecyl sulfate polyacrylamide gel electrophoresis gel and transferred onto a polyvinylidene difluoride membrane. The membrane was blocked with 5% non-fat milk in PBS and incubated overnight at 4°C with one of the following primary antibodies: Rabbit polyclonal anti-SIRT1 (1:400; Santa Cruz Biotechnology, Inc.); rat polyclonal anti-insulin receptor (InsR; 1:1,000; Wuhan Boster Biological Technology, Ltd., Wuhan, China); rabbit polyclonal anti-glucose transporter 2 (Glut2, 1:400; Santa Cruz Biotechnology, Inc.); rabbit polyclonal anti-phospho-Tyr467-phosphatidylinositol-4,5-bisphosphate 3-kinase (PI3K; 1:400; Santa Cruz Biotechnology, Inc.); rabbit polyclonal anti-total-PI3K (1:1,000; Wuhan Boster Biological Technology, Ltd.); rabbit polyclonal anti-phospho-Ser473-AKT (1:400; Santa Cruz Biotechnology, Inc.); rabbit polyclonal anti-total-AKT (1:400; Wuhan Boster Biological Technology, Ltd.). Following incubation with the primary antibody, the membranes were washed and incubated with horseradish peroxidase-conjugated secondary antibodies at 37°C for 45 min. Enhanced chemiluminescence reagent (Millipore) was used for detection of protein bands. The densities of the protein bands were determined using a gel imaging system (Beijing Sixty-One Instrument, Beijing Six-One Instrument, Beijing, China). The expression levels of Sirt1, InsR, total PI3K, phosphorylated-PI3K, total AKT, phosphorylated-AKT and Glut2 protein levels were normalized against those of β-actin levels.

### Statistical analysis

Data are presented as the mean ± standard deviation. For a comparison between two groups, Student’s t-test was used. For comparisons among three or more groups, one-way analysis of variance was used. P<0.05 was considered to indicate a statistically significant difference.

## Results

### RSV suppresses CIH-induced body weight gain in rats

**Figure 1:**
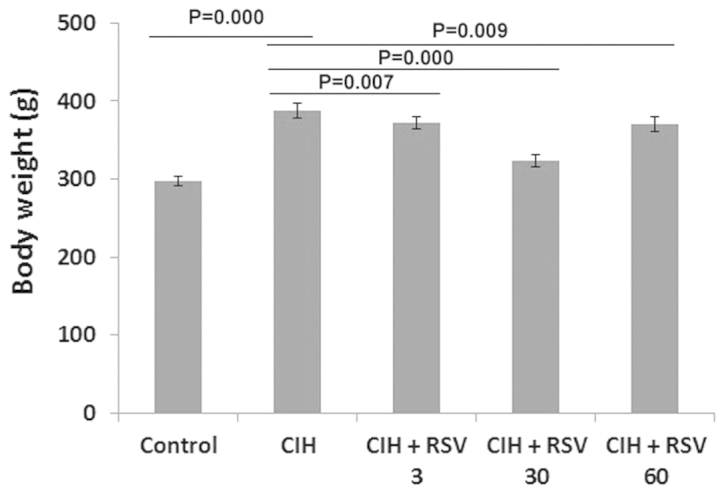
Body weights of rats. Rats were treated daily with or without RSV (3, 30 or 60 mg/kg/day) via an oral gavage. Rats were placed daily in intermittent hypoxic chambers for 8 h each day for 28 days. Control rats were placed in normoxic chambers. On day 29, following the treatment, the body weights of rats (n=8 in each group) were recorded. Compared with the control group, the body weights of rats significantly increased in the CIH-treated groups (P=0.000). Compared with the CIH group, the body weights of rats significantly decreased in the CIH-treated groups following RSV treatment (all P<0.001). RSV, resveratrol; CIH, chronic intermittent hypoxia.

Weight gain is associated with chronic hypoxia in animals and humans. To evaluate the effect of RSV on body weight in animals following hypoxia exposure, the weight of each animal was recorded every week. Prior to exposure to hypoxia, all rats had similar body weights. Compared with the control group, the body weight in rats significantly increased following CIH exposure. At day 29, the average body weight of control rats was 297 g and that of CIH-treated rats was 388 g (P=0.000). The body weights in rats treated with RSV decreased; the average weight was 372.125 g for the CIH plus low RSV group, 323.675 g for the CIH plus median RSV group and 370.250 g for the CIH plus high RSV group. Compared with the CIH group, there were significant differences among RSV-treated groups (P<0.01). These results demonstrate that CIH increases the body weight of rats, however, RSV inhibits this weight gain (Fig. 1).

### RSV reverses CIH-induced IR in rats

**Figure 2:**
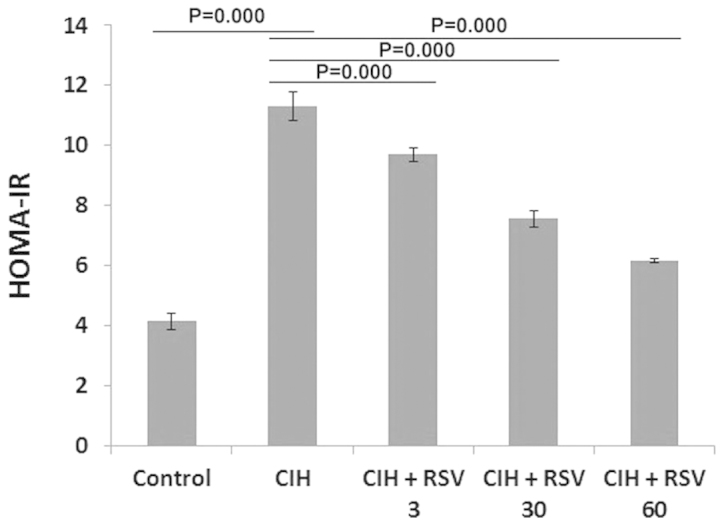
HOMA-IR index in rats. Rats were treated daily with or without RSV (3, 30 or 60 mg/kg/day) via an oral gavage. Rats were placed daily in intermittent hypoxic chambers for 8 h each day for 28 days. Control rats were placed in normoxic chambers. On day 29 following the treatment, blood was collected from all animals. Blood glucose levels were measured using a Contour glucometer. Blood insulin levels were measured using a rat insulin ELISA kit. The HOMA-IR index was calculated using the following formula: [Fasting insulin concentration (μU/ml) × fasting glucose concentration (mmol/l)] / 22.5, assuming that normal subjects have an insulin resistance of 1. CIH increased the HOMA-IR in rats (n=8 for each group) and RSV significantly reversed this effect. The HOMA-IR index significantly increased in the CIH-exposed group compared with the control group (P=0.000) and significantly decreased in RSV-treated groups (P=0.000). RSV, resveratrol; CIH, chronic intermittent hypoxia; HOMA-IR, homeostasis model assessment-estimated insulin resistance.

To evaluate the effect of RSV on IR following chronic hypoxia exposure, the blood glucose and insulin levels of animals was measured on day 29 following hypoxia exposure. IR was assessed using the HOMA-IR index. Compared with the control group, the HOMA-IR values of CIH-treated groups were significantly higher (P=0.000). The values were 4.14±0.29 (control group), 11.32±0.47 (CIH group), 9.70±0.24 (CIH plus low RSV group), 7.55±0.29 (CIH plus median RSV group) and 6.16±0.06 (CIH plus high RSV group), respectively. Compared with the CIH without RSV group, the values significantly decreased in other CIH-treated groups following RSV treatment (P=0.000). The reduction in RSV-treated groups was dose dependent. However, the values in CIH-exposed groups remained higher than that observed in the control group regardless of RSV treatment (Fig. 2).

### RSV protects rats against CIH-induced liver damage

**Figure 3:**
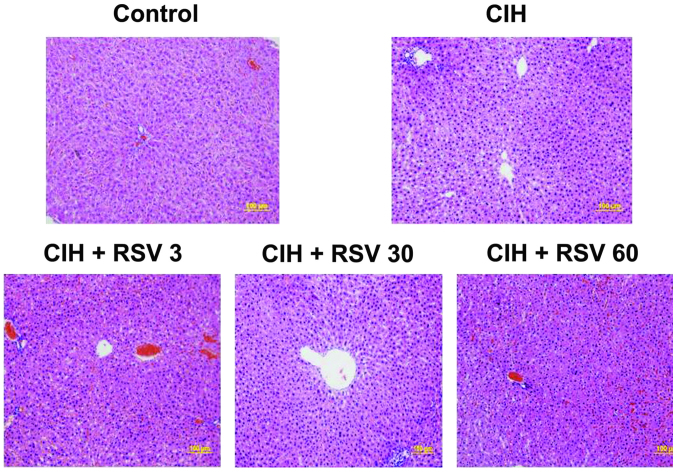
Morphology of liver tissue in rats. Rats were treated daily with or without RSV (3, 30 or 60 mg/kg/day) via an oral gavage. Rats were placed daily in intermittent hypoxic chambers for 8 h each day for 28 days. Control rats were placed in normoxic chambers. On day 29 following the treatment, animals were sacrificed in a postprandial state under isoflurane anesthesia. Livers were collected for histological analysis. Liver tissue was routinely fixed and embedded in paraffin blocks. Paraffin-embedded tissues (4 μm thick) were sectioned and stained with hematoxylin and eosin (magnification, ×100). RSV, resveratrol; CIH, chronic intermittent hypoxia.

To evaluate the morphological alterations in the rat liver following CIH exposure and the effect of RSV on these changes, liver tissue sections were stained with H&E. The hepatocytes in the control rat tissue were tightly arranged together with clearly visible nuclei. The hepatocytes in the CIH-treated rats were swollen accompanied with nuclear pyknosis, degeneration and only partial cytoplasmic vacuoles were present. The changes in the group treated with a low dose of RSV were similar to that observed in the CIH without RSV group, for example, swollen hepatocytes, fuzzy cell boundaries, nuclear pyknosis and the presence of partial cytoplasmic vacuole were observed. The cell boundaries in the median RSV treated rats were less fuzzy than those observed in the control group. The changes in the group treated with a high dose of RSV appeared normal. These observations suggested that RSV may protect the rat against CIH-induced liver damage (Fig. 3).

### RSV upregulates the mRNA and protein levels of Sirt1 in the liver

**Figure 4:**
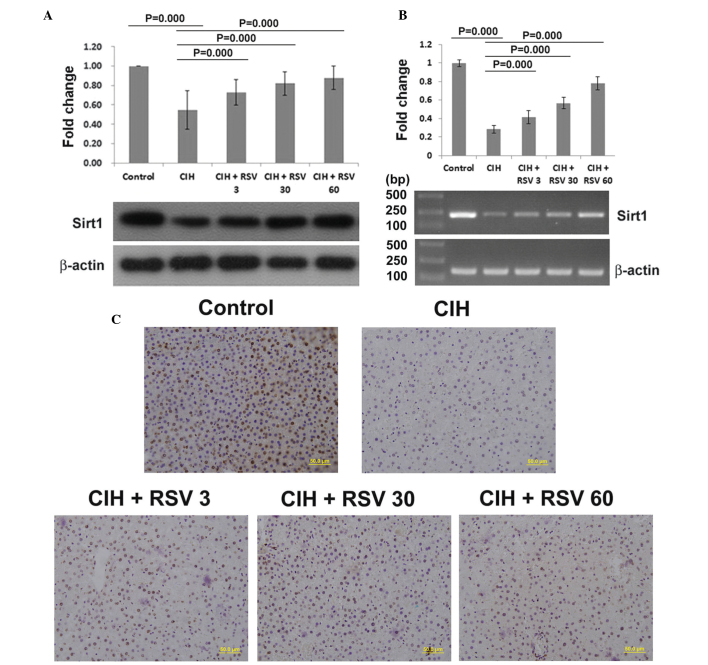
Sirt1 levels in rat liver. Rats were treated daily with or without RSV (3, 30 or 60 mg/kg/day) via an oral gavage. Rats were placed daily in intermittent hypoxic chambers for 8 h each day for 28 days. Control rats were placed in normoxic chambers. On day 29 following the treatment, rat liver was harvested. (A) Proteins from hepatic tissue were extracted, resolved on a 10% SDS-PAGE gel, transferred onto a membrane and stained with antibody against Sirt1. (B) Relative mRNA levels of *SIRT1* in the liver were measured by quantitative polymerase chain reaction. β-actin was used as an internal control. Sirt1 protein and mRNA measurements were normalized to levels of β-actin protein and mRNA, respectively, and expressed as fold-change. (C) Paraffin-embedded hepatic tissue sections were stained with antibody against Sirt1 for immunohistochemistry analysis as described in Materials and methods. mRNA and protein levels of Sirt1 significantly decreased in the CIH group compared with that in the control group (P=0.000). It significantly increased in RSV-treated groups (all P=0.000). RSV, resveratrol; CIH, chronic intermittent hypoxia.

RSV is hypothesized to be the most potent activator of Sirt1. To evaluate the effect of RSV on Sirt1 levels in liver following CIH exposure, mRNA and protein levels of Sirt1 in liver tissue were analyzed. Compared with the control group, Sirt1 protein levels in CIH-treated groups significantly reduced following CIH exposure (P=0.000). However, the levels in the RSV-treated CIH groups were elevated compared with that in the CIH group without RSV treatment (P=0.000). These changes were dose dependent (Fig. 4A). The mRNA changes of *SIRT1* were similar to that observed in the protein levels. *SIRT1* mRNA levels in the hepatic tissue were significantly reduced following CIH (P=0.000), however, RSV treatment significantly increased the mRNA levels (P=0.000; Fig. 4B). The IHC staining revealed a significant reduction in the SIRT1-positive cells in hepatic tissue sections from rats subjected to CIH compared with that detected in the control group, however, RSV treatment led to an increase in Sirt1-positive cells (Fig. 4C). These results suggested that RSV significantly upregulated the levels of Sirt1 in rats following CIH exposure.

### RSV downregulates InsR and Glut2 levels in the liver

**Figure 5:**
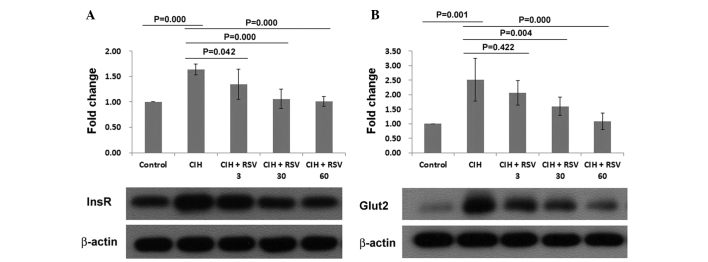
Liver protein levels of InsR and Glut2 in rats. Rats were treated daily with or without RSV (3, 30 or 60 mg/kg/day) via an oral gavage. Rats were placed daily in intermittent hypoxic chambers for 8 h each day for 28 days. Control rats were placed in normoxic chambers. On day 29 following the treatment, rats were sacrificed and liver was harvested. Proteins from hepatic tissue were harvested, resolved on a 10% SDS-PAGE gel and immunostained with antibody against (A) InsR and (B) Glut2. InsR and Glut2 levels were normalized to levels of β-actin protein and were expressed as fold change. InsR and Glut2 protein levels significantly increased in the CIH group compared with the control group. The levels decreased in RSV-treated groups. RSV, resveratrol; CIH, chronic intermittent hypoxia; InsR, insulin receptor; Glut2, glucose transporter 2.

InsR and Glut2 are important in regulating glucose metabolism. The levels are positively correlated with IR and diabetes. To evaluate the effect of RSV on these levels in the liver, the levels of the two proteins were analyzed using western blotting. The gel densities were quantitated and expressed as fold-changes following normalizing against the levels of β-actin. The relative protein InsR level was significantly higher in the CIH group compared with that in the control group (P=0.000; Fig. 5A). Compared with the CIH without RSV group, RSV treatment significantly reduced InsR levels (P=0.042, 3 mg/kg/day RSV; P=0.000, 30 mg/kg/day RSV; P=0.000, 60 mg/kg/day RSV). The level of InsR in the group treated with a low dose of RSV was higher than that in the normal control, while, the level of InsR in the groups treated with a median and high dose of RSV were similar to that in the normal control (Fig. 5A). The alterations in Glut2 levels were similar to those of InsR. Glut2 levels significantly increased in the CIH group compared with that in the control group (P=0.000). Compared with the CIH without RSV group, RSV treatments significantly reduced Glut2 levels at 30 and 60 mg/kg/day of RSV, but not 3 mg/kg/day of RSV (P=0.422, 3 mg/kg/day RSV; P=0.004, 30 mg/kg/day RSV; P=0.000, 60 mg/kg/day RSV; Fig. 5B).

### RSV activates phosphorylation of PI3K and AKT in the liver

**Figure 6:**
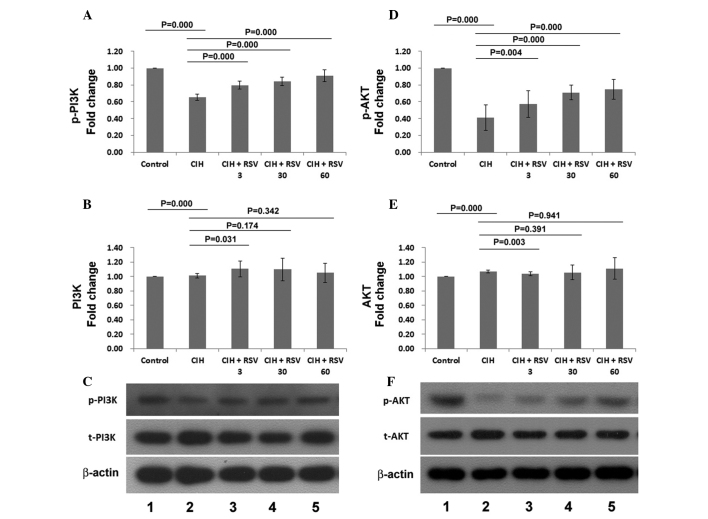
Phosphorylation of PI3K and AKT in rat liver. Rats were treated daily with or without RSV (3, 30 or 60 mg/kg/day) via an oral gavage. Rats were placed daily in intermittent hypoxic chambers for 8 h each day for 28 days. Control rats were placed in normoxic chambers. On day 29 following the treatment rats were sacrificed and hepatic tissue was harvested. Proteins from hepatic tissue were extracted, resolved on a 10% SDS-PAGE gel, transferred onto a membrane and stained with antibodies against (A and C) phospho-Tyr467-PI3K, (B and C) t-PI3K and (D and F) phospho-Ser473-AKT and (E and F) t-AKT. Protein measurements were normalized to levels of β-actin protein in each group and were expressed as fold change. p-PI3K, phosphorylated-PI3K; t-PI3K, total PI3K; p-AKT, phosphorylated-AKT; t-AKT, total AKT; RSV, resveratrol; CIH, chronic intermittent hypoxia; PI3K, phosphatidylinositol-4,5-bisphosphate 3-kinase.

To further investigate the underlying mechanisms by which RSV reduces IR in chronic hypoxic animals, the phosphorylation of PI3K and AKT was analyzed. The data demonstrated that CIH exposure significantly inhibited the phosphorylation of PI3K and AKT (P=0.000). RSV treatment significantly upregulated the phosphorylation of PI3K and AKT (P=0.000). RSV treatment had no effect on total PI3K and total AKT protein levels following CIH exposure (P>0.05) with the exception of 3 mg/kg/day RSV (P<0.05). Compared with the control group, the total levels of PI3K and AKT in the CIH group increased (P=0.000; Fig. 6).

## Discussion

Sleep apnea is considered a major cause of obesity and diabetes in patients suffering from this disorder. Patients with OSA undergo repeated cycles of intermittent hypoxia during sleep. In the present study, rats were exposed to a chronic intermittent hypoxic environment to mimic the episodic hypoxia occurring in patients with OSA. The major findings included: i) CIH increased the body weights of animals and caused liver damage; ii) CIH induced IR in animals; iii) RSV reversed IR and improved the structure of the liver; iv) Upregulation of Sirt1 and activation of PI3K/AKT signaling are possibly involved in this process.

Chronic hypoxia is often associated with weight gain and obesity. The present study demonstrated that the body weights in CIH-treated animals significantly increased. RSV was able to prevent the weight gain in rats. This effect varied under different doses of RSV. Low and high doses of RSV partially prevented the weight gain, however, a medium dose of RSV significantly prevented the weight gain. The reasons for this difference remain to be elucidated. In addition to preventing body weight gain, RSV can protect rats against CIH-induced liver damage. The high dose of RSV offered optimal protection against hepatic damage.

Episodic hypoxemia poses a severe threat to energy metabolism and glucose homeostasis. In the present study, the HOMA-IR index, which reflected IR, increased significantly in animals exposed to CIH. This suggests that CIH induces significant IR, which leads to decreased blood insulin levels and increased blood glucose levels. RSV treatment significantly reversed IR induced by CIH in rats. This effect was dose dependent. It was also noted that CIH significantly increased InsR and Glut2 protein levels in rat hepatic tissues. The levels were significantly reduced with RSV treatment in a dose-dependent manner. These results also suggest that RSV was effective in ameliorating IR induced by CIH and also enhanced insulin sensitivity *in vivo*. The present results are consistent with previous studies (27–32).

RSV has been suggested as a potent activator of Sirt1. The effects of RSV on metabolism, cancer, aging and inflammation were revealed through upregulation of Sirt1 (12,34). Sirt1 is important in these biological processes (35–37). The importance of Sirt1 activation in energy metabolism has been confirmed in *SIRT1*-knockout mice as well as mice that overexpress *SIRT1*. In the present study, it was demonstrated that CIH decreased hepatic Sirt1 expression. RSV upregulated liver Sirt1 expression at the translational and transcriptional levels. It is postulated that activation of Sirt1 may be involved in the process by which RSV exerts its effect in rats, however, further investigation is required.

Insulin stimulates glucose uptake and its metabolism in the peripheral tissues. The insulin signaling pathway is fundamental in regulating blood insulin levels and maintaining glucose homeostasis. This pathway involves a series of signaling cascades that are activated by binding of insulin to its receptor, followed by autophosphorylation of the InsR, activation of the receptor tyrosine kinase and tyrosine phosphorylation of InsR substrates (IRSs). Phosphorylation of IRSs leads to activation of PI3K and subsequently to activation of serine/threonine kinase, AKT - a downstream mediator of PI3K signaling (26,39). The PI3K/AKT pathway is a key component of the insulin signaling cascade and is considered necessary for glucose transport (38). Previous studies have demonstrated that insulin-stimulated PI3K activity decreases in the skeletal muscles of patients with type II diabetes (40,41). In the current study, phosphorylation of PI3K and AKT decreased in the hepatic tissue following CIH exposure. RSV treatment significantly increased the fraction of phosphorylated PI3K and partially increased the fraction of phosphorylated AKT in the hepatic tissue. RSV also partially increased the total protein levels of PI3K and AKT in the hepatic tissue. These findings suggest that activation of the PI3K/AKT signaling pathway may contribute to the effects of RSV as demonstrated in the present study.

In conclusion, the current study has demonstrated that RSV effectively reverses IR in rats, which was induced by chronic hypoxia. Upregulation of Sirt1 and activation of PI3K/AKT signaling may be involved in this process. Without loss of function studies, it is difficult to form conclusions. Previous studies have reported that Sirt1 modulates insulin signaling by interacting with p85, the PI3K adapter subunit, in an insulin-independent manner (42). The direct interaction between Sirt1 and PI3K in regulating insulin signaling was not investigated in the present study. This is an important question, which is to be elucidated in future studies.
